# Silk fibroin and ceramic scaffolds: Comparative in vitro studies for bone regeneration

**DOI:** 10.1002/btm2.10221

**Published:** 2021-04-08

**Authors:** Rucha Deshpande, Swati Shukla, Raeesa Sayyad, Shalmali Salunke, Anuya Nisal, Premnath Venugopalan

**Affiliations:** ^1^ Research and Development Serigen Mediproducts Pvt. Ltd. Satara Road Pune Maharashtra India; ^2^ Department of Polymer Science and Engineering, CSIR‐National Chemical Laboratory Pune India

**Keywords:** beta tricalcium phosphate, bone regeneration, bone void filler/scaffold, calcium sulphate, hMSCs, hydroxyapatite, osteoblasts, silk fibroin

## Abstract

Synthetic bone void fillers based on calcium ceramics are used to fill cavities in the bone and promote bone regeneration. More recently, silk fibroin (SF), a protein polymer obtained from *Bombyx mori* silkworm, has emerged as a promising material in bone void filling. In this work, we have compared the safety and efficacy of two types of silk fibroin‐based bone void fillers with currently used and commercially available ceramic bone void fillers (based on calcium sulphate, beta tricalcium phosphate, and beta tricalcium phosphate with hydroxyapatite). Further, we have also evaluated these two types of SF scaffolds, which have strikingly different structural attributes. The biocompatibility of these scaffolds was comparable as assessed by cytotoxicity assay, cellular adhesion assay, and immunogenic assay. Ability of the scaffolds to support differentiation of human mesenchymal stem cells (hMSCs) into an osteoblastic lineage was also evaluated in an in vitro differentiation experiment using reverse transcriptase polymerase chain reaction analysis. These results revealed that cells cultured on SF scaffolds exhibit higher expression of early to late markers such as Runx2, BMPs, collagen, osterix, osteopontin, and osteocalcin as compared with ceramic‐based scaffolds. This observation was further validated by studying the expression of alkaline phosphatase and calcium deposition. We also show that scaffolds made from same material of SF, but characterized by very different pore architectures, have diverse outcome in stem cell differentiation.

**List of Abbreviations**
ALP: alkaline phosphatase
β‐TCP: beta tricalcium phosphate
BMP: bone morphogenic protein
CaSO_4_
: calcium sulphate
DMSO: dimethyl sulphoxide
HA: hydroxyapatite
HDPE: high‐density polyethylene
hMSC: human mesenchymal stem cells
IL‐1β: interleukin 1 beta
LPS: lipopolysaccharide
L‐RSF: lyophilized‐regenerated silk fibroin
M‐RSF: microparticle‐regenerated silk fibroin
MPa: mega Pascal
OCN: osteocalcin
OPN: osteopontin
RT‐PCR: reverse transcriptase polymerase chain reaction
SF: silk fibroin
TNF‐α: tumor necrosis factor alpha

## 1 INTRODUCTION

The global synthetic bone void filling market was pegged at ~US$2.8 billion in 2019 and is expected to grow at a cumulative annual growth rate  of > 5% over the period of 2018–2028.^1^ Bone void fillers or bone grafts are materials that are used to fill defects or cavities formed in the bone. Bone cavities may be formed due to accidents or may also be caused due to a tumor or an infection in the bone. To accelerate the healing of bone in these clinical conditions, the cavities are typically filled with a bone void filler. A variety of natural and synthetic materials have been used as bone void fillers. More than 50% of the materials used for bone void filling are based on calcium ceramics. These include materials such as hydroxyapatite (HA), calcium sulphate (CaSO_4_), beta tricalcium phosphate (β‐TCP), and their composites/blends.^2^, ^3^, ^4^, ^5^, ^6^, ^7^

HA has been extensively used for bone void filling applications for several decades. It is biocompatible and bioinert, that is, it does not induce an inflammatory response from the host tissue and is generally well tolerated at the implantation site. It has also been shown to support new bone formation. However, HA has an extremely slow rate of bioresorption and remains at the implantation site for several years. This has been a cause for concern and more recently other alternatives are being evaluated for this application. β‐TCP‐ and CaSO_4_‐based bone void fillers overcome this problem of slow resorption of HA. These materials have been demonstrated to also exhibit excellent support for the bone regeneration. However, both β‐TCP and CaSO_4_ have extremely fast rates of bioresorption and are found to resorb within a few months after implantation.^7^, ^8^ This faster rate of resorption results in other complications such as incomplete filling of defects, poor quality of new bone formation, and sometimes also secondary fractures.^6^, ^9^, ^10^ Thus, there is an active interest to develop novel bone void filling materials that overcome these limitations of existing materials.

Silk fibroin (SF), a natural polymer extracted from the *Bombyx mori* silkworm cocoon, has been explored as a promising material for bone void filling applications.^11^ SF has exceptional thermomechanical properties, inherent and proven biocompatibility, easy processability, and controlled rate of bioresorption. Several researchers have demonstrated innovative processing protocols to make scaffolds of SF and have shown that these materials support new bone formation.^12^, ^13^, ^14^ SF has also been blended with other biopolymers and bio‐ceramics and these composites have also shown promising results in bone regeneration.^13^ These scaffolds produced from SF and its blends/composites have varying porosities, pore architectures, and pore sizes. The scaffolds also exhibit a broad range of mechanical properties—for example, compression modulus varying from 0.1 MPa to >50 MPa. The conformation of the SF protein can also be controlled using various physical and chemical treatments and it has been shown to affect the mechanical and bioresorption characteristics of the scaffold.^15^

In spite of the large volume of literature on SF and ceramics scaffolds for bone regeneration, there are not many studies that compare and contrast the ability of these scaffolds to support bone regeneration. Thus, the objective of this study is twofold. The first objective is to compare the ability of SF scaffolds vis‐a‐vis calcium‐based ceramic scaffolds to support bone regeneration. This was done using in vitro assays that monitored the differentiation markers of new bone formation. The second objective is to compare the performance within two silk scaffolds, which have strikingly different structural attributes in bone regeneration. We selected three representative commercial ceramic bone void fillers that had a chemical composition consisting of CaSO_4,_ ^16^ β‐TCP,^17^ and a composite of β‐TCP‐HA.^18^ These materials were selected since they are commercially available globally and are preferred products by several clinicians performing bone void filling surgeries. Further, we prepared two different types of SF scaffolds. An SF microparticle‐based scaffold was prepared as per the protocol described in the study by Nisal et al. The scaffold exhibited low porosity and high compression modulus of ~70 MPa in dry state and ~18 MPa in wet state.^19^ A second SF scaffold was prepared by lyophilizing regenerated SF solution. This scaffold had high porosity (>90%) and relatively lower mechanical properties (compression modulus ~10 MPa in dry state and ~3 MPa in wet state). Our work indicates that SF scaffolds, designed with large pore size (>100 μm) and pore interconnectivity, appropriate bulk porosity (>40%) and excellent mechanical properties (wet compression modulus > 18 MPa), supports better bone regeneration as compared to ceramic‐based scaffolds.

## 2 RESULTS

Here, we compare the performance of the SF scaffolds vis‐à‐vis the conventionally used and commercially available calcium‐based ceramic bone void fillers in bone regeneration using in vitro techniques. Further, we also used two types of SF scaffolds lyophilized‐regenerated silk fibroin (L‐RSF) and microparticle‐regenerated silk fibroin (M‐RSF), with significantly different mechanical and structural properties.

The bioceramic, L‐RSF and M‐RSF scaffolds vary in their pore size, porosity, and mechanical performance and crystallinity index. These properties were measured using standard protocols described in detail in our earlier manuscript Nisal et al. and have been tabulated in Table 1.^19^ The properties have also been discussed in Section 4 of the manuscript. Photographs of all scaffolds used in the study are included in Figure S1A. The bioceramic materials were used as is or as per the protocols described by the manufacturer. The material of construction of both silk scaffolds (L‐RSF and M‐RSF) is a natural protein polymer—SF. L‐RSF scaffolds have significantly higher porosity as compared to M‐RSF. L‐RSF scaffold has random pores as shown in Figure S1B. Compression modulus of L‐RSF scaffolds is significantly lower than M‐RSF (Table 1).

**TABLE 1:** Comparative analysis of ceramic‐based scaffolds (CaSO_4_, β‐TCP, and β‐TCP‐HA) with silk‐based scaffold (L‐RSF and M‐RSF)

		Ceramic scaffolds			Silk scaffolds	
Property		CaSO_4_	β‐TCP	β‐TCP‐HA	L‐RSF	M‐RSF
Compression modulus		∼80 MPa (dry) ∼70 MPa (wet)	∼5 MPa	Not available	∼10 MPa (dry) ∼3 MPa (wet)	∼70 MPa (dry) ∼18 MPa (wet)
Porosity		10%–12%	60%–70%	60%–70%	90%–95%	40%–44%
Pore size		Randomly packed crystals < 5 μm	100–500 μm (macropores) ≤10 μm (micropores)	300–600 μm (macropores) ≤10 μm (micropores)	10–200 μm (random pores)	0–275 μm
% Cellular adhesion		92.2 ± 1.5	94.3 ± 1.2	91.4 ± 2.3	93.7 ± 2.2	88.7 ± 2.2
Proliferation (A 570 nm)		0.27 ± 0.03	0.41 ± 0.02	0.36 ± 0.06	0.48 ± 0.03	0.90 ± 0.07
Early markers^a^	Runx‐2	0.96 ± 0.11	0.99 ± 0.10	0.58 ± 0.06	1.2 ± 0.16	1.2 ± 0.09
	Osterix	0.85 ± 0.06	0.78 ± 0.11	0.48 ± 0.18	1.27 ± 0.14	1.5 ± 0.08
	BMP‐2	0.53 ± 0.09	0.48 ± 0.06	0.21 ± 0.10	1.77 ± 0.16	2.49 ± 0.09
Early to late markers^a^	Col1α1	0.33 ± 0.05	0.31 ± 0.03	0.13 ± 0.04	1.54 ± 0.11	2.92 ± 0.09
	BMP‐4	0.57 ± 0.04	0.81 ± 0.24	0.40 ± 0.04	1.31 ± 0.07	1.46 ± 0.11
	BMP‐6	0.74 ± 0.09	0.66 ± 0.13	0.25 ± 0.06	1.89 ± 0.06	2.35 ± 0.09
Late markers^a^	OPN	0.63 ± 0.04	0.61 ± 0.15	0.47 ± 0.11	1.51 ± 0.10	2.91 ± 0.09
	OCN	0.95 ± 0.06	0.86 ± 0.11	0.61 ± 0.10	1.47 ± 0.10	1.66 ± 0.09
ALP activity (ALP activity/100 μg of protein)		8.31 ± 0.65	11.18 ± 0.39	9.81 ± 0.13	14.98 ± 1.32	22.17 ± 1.42
Ca^2+^ deposition (A 405 nm)		1.31 ± 0.20	1.63 ± 0.16	1.79 ± 0.23	1.93 ± 0.12	2.69 ± 0.35

Abbreviations: ALP, alkaline phosphatase; CaSO_4_, calcium sulphate; β‐TCP, beta tricalcium phosphate; β‐TCP‐HA, beta tricalcium phosphate with hydroxyapatite; L‐RSF, lyophilized‐regenerated silk fibroin; M‐RSF, microparticle‐regenerated silk fibroin.

a Level of marker expression is expressed in fold difference in (mean fluorescence intensity (M.F.I.).

The in vitro studies were conducted at two levels, assessment of biocompatibility and assessment of efficacy in supporting differentiation of human mesenchymal stem cells (hMSCs) to osteoblasts.

### 2.1 Biocompatibility testing

#### 2.1.1 Cytotoxicity testing

In vitro cytotoxicity testing was done as described in ISO‐10993 to evaluate the overall biocompatibility and safety of the biomaterials. This was carried out using both direct contact and extract contact methods. Organo‐Tin PU is a known cytotoxic material and hence used as a positive control and HDPE, known to be nontoxic was used as negative control for cytotoxicity, for direct contact method.

**FIGURE 1:**
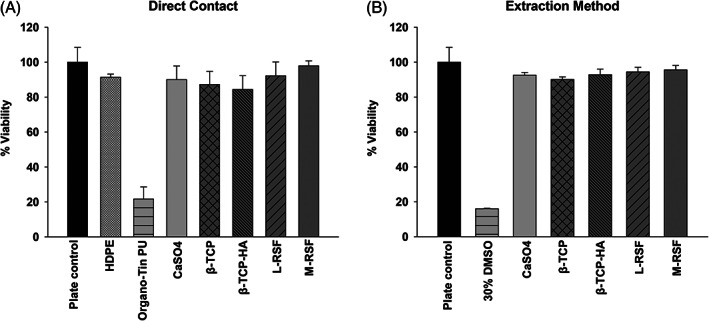
Cytotoxicity assay: (a) Direct contact method: L929 cells (10,000 cells/well) were seeded on 96‐well plate and incubated for 24 h at 37°C, 5% CO_2_ atmosphere. After 24 h of incubation, media were replaced and scaffolds were placed in direct contact with cells (calcium sulphate [CaSO_4_], beta tricalcium phosphate [β‐TCP], beta tricalcium phosphate with hydroxyapatite [β‐TCP‐HA], lyophilized‐regenerated silk fibroin [L‐RSF] scaffold, and microparticle‐regenerated silk fibroin [M‐RSF]), high‐density polyethylene (HDPE) (negative control) and Organo‐tin PU (positive control). Plates were incubated further for 24 h. (b) Extraction method: L929 cells were seeded as described above. After 24 h of incubation cells, media were replaced with complete medium (negative control), 30% DMSO (positive control) and extract of all test and control scaffolds. Cells were further incubated for 24 h. After 24 h of treatment MTT proliferation assay was performed and % viability was calculated. Data from three independent experiments are represented

Extracts of these materials were also assessed for cytotoxicity by 3‐(4,5‐dimethylthiazol‐2‐yl)‐2,5‐ diphenyltetrazolium bromide (MTT) assay. Results of cytotoxicity testing using direct contact and extraction method are summarized in Figure 1(a,b), respectively. L929 cells in contact with both silk scaffolds (L‐RSF and M‐RSF) displayed comparable viability with plate control, all benchmarking products and negative control (HDPE) (Figure 1(a)). Cell viability for Organo‐tin PU sample was found to be <25% and this confirmed the validity of the experiment. Extracts of both silk scaffolds (L‐RSF and M‐RSF) exhibited >90% viability (Figure 1(b)). These results indicate that both L‐RSF and M‐RSF scaffolds or their extracts are not cytotoxic. Also, the viability of cells in both studies is at par with the commercial products used for benchmarking.

#### 2.1.2 Assessment of cellular adhesion

**FIGURE 2:**
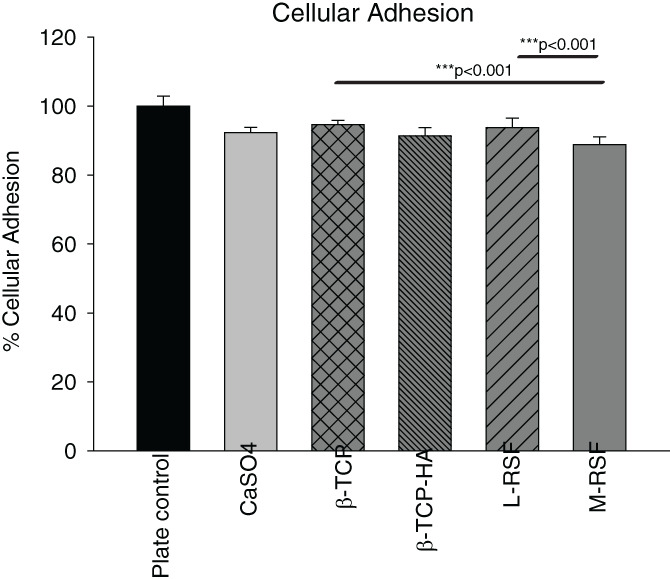
Cellular adhesion assay. Equal number of L929 cells were seeded on 96‐well plate (plate control), calcium sulphate (CaSO_4_), beta tricalcium phosphate (β‐TCP), beta tricalcium phosphate with hydroxyapatite (β‐TCP‐HA), lyophilized‐regenerated silk fibroin (L‐RSF) scaffolds and microparticle‐regenerated silk fibroin (M‐RSF), cells were incubated for 24 h at 37°C, 5% CO_2_ atmosphere. After 24 h, cells were stained with 2% crystal violet solution for 30 min. Cells were then washed with PBS and crystal violet was extracted in 5% SDS. Absorbance was measured at 595 nm. Data normalized with plate control and expressed as average percent cellular adhesion ± SD

Cellular adhesion to matrix plays an integral role in cell‐scaffold interaction as well as cell–cell communication and is of vital importance in the development and maintenance of tissue. Anchoring of cells onto the substrate is essential for stimulating signals that regulate cell viability, proliferation, and differentiation.^20^, ^21^ Thus, in vitro evaluation of affinity of cells to substrate is of crucial importance in the development of bio‐scaffolds. Hence, we tested cellular adhesion on M‐RSF, L‐RSF scaffolds, CaSO_4_, β‐TCP, and β‐TCP‐HA. Results of cellular adhesion are summarized in Figure 2. It was observed that ~90% cells adhere to all scaffolds, indicating that all these materials do support cellular adhesion to a similar extent. Within the silk scaffolds, L‐RSF scaffolds showed marginally better cell adhesion than M‐RSF. However, when the silk scaffolds were compared with the ceramic‐based material, M‐RSF showed comparable cellular adhesion with CaSO_4_ and β‐TCP‐HA, but marginally lower cellular adhesion was seen when compared to β‐TCP.

#### 2.1.3 Evaluation of inflammatory response^:^

**FIGURE 3:**
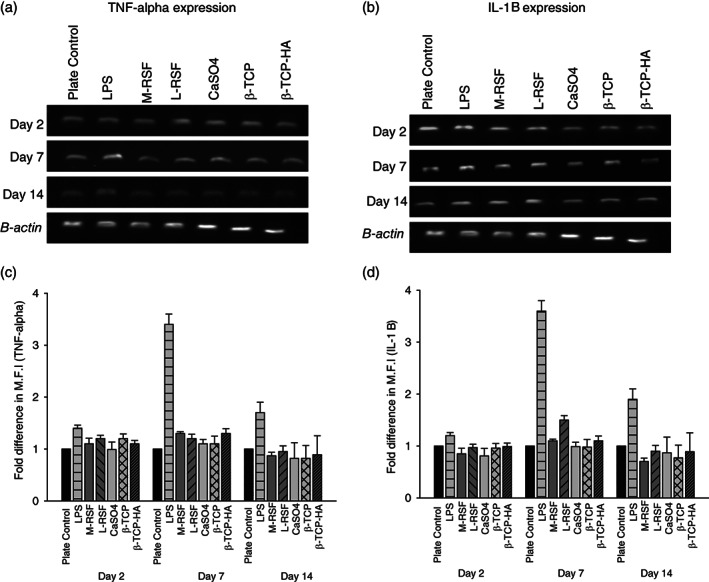
Assessment of inflammatory response: RAW 264.7 cells were cultured in presence of scaffolds for 14 days at 37°C in 5% CO_2_ atmosphere. Inflammatory response was assessed at Days 2, 7, and 14 by measuring expression levels of tumor necrosis factor alpha (TNF‐α) and interleukin 1 beta (IL‐1β). Cells treated with lipopolysaccharide (LPS) (1 mg/ml) and untreated wells were used as positive and negative controls, respectively. At time points Days 2, 7, and 14 mRNA were extracted from cells, converted into cDNA and amplified using TNF‐α and IL‐1β specific primers. Panels (a) and (c) show reverse transcriptase polymerase chain reaction (RT‐PCR) gel image of TNF‐α and IL‐1 β, respectively. (b) and (d) are densitometric analysis of band intensities of TNF‐α and IL‐1 β expression, was done by Image J software. Fold difference in mean fluorescence intensity (M.F.I.) was plotted

Inflammation is the first physiological response upon implantation and hence it is necessary to assess the inflammatory response of any implantable material. An assay based on mouse macrophage cell line RAW 264.7 was used. A negative control in the form of tissue culture plate was incorporated in the experiment. Lipopolysaccharide (LPS) is known to induce inflammatory response and was therefore included as a positive control. All scaffolds were exposed to a monolayer of macrophage cells for defined time intervals, to allow cells to elicit immune response. Tumor necrosis factor alpha (TNF‐α) and interleukin 1 beta (IL‐1β) levels were quantified on days 2, 7, and 14 and the results are summarized in Figure 3. Figure 3(a,b) shows agarose gel images of TNF‐α and IL1β PCR, respectively, and their respective densitometric analyses (Figure 3(c,d)). LPS‐treated cells showed expected induction of both cytokines and untreated cells showed only the basal level of expression indicating the validity of experiment. No significant difference in TNF‐α and IL‐1β expression among all scaffolds and plate control was observed, on Days 2, 7, and 14 confirming noninflammatory nature of these scaffolds. TNF‐α and IL‐1β levels in LPS‐treated cells were approximately twofold higher than that of plate control. These results also corroborate with earlier reports where biocompatibility of SF is proven for tissue engineering applications.^22^, ^23^, ^24^

### 2.2 Efficacy of scaffolds in supporting differentiation of hMSC's into osteoblasts (bone cells)

**FIGURE 4:**
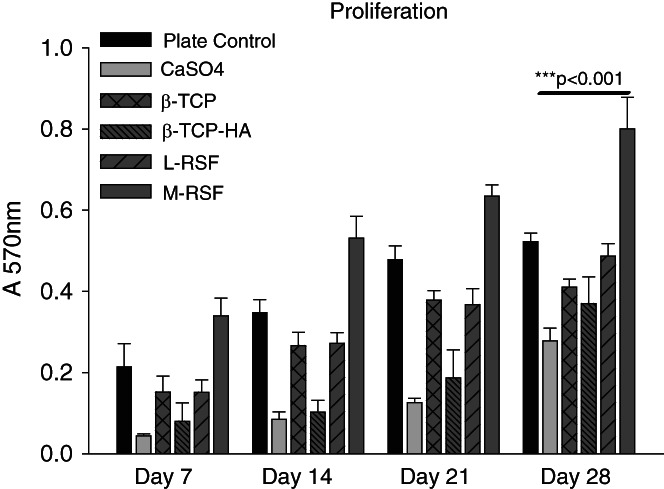
Proliferation of human mesenchymal stem cells (hMSCs) on all scaffolds: hMSCs were seeded on calcium sulphate (CaSO_4_), beta tricalcium phosphate (β‐TCP), beta tricalcium phosphate with hydroxyapatite (β‐TCP‐HA), lyophilized‐regenerated silk fibroin (L‐RSF), and microparticle‐regenerated silk fibroin (M‐RSF). Cells were cultured in osteogenic media and incubated at 37°C, 5% CO_2_ for 28 days with media change at every 48 h. Proliferation was measured at Days 7, 14, 21, and 28. Data expressed as mean ± SD (*N* = 3) ***p < 0.001

**FIGURE 5:**
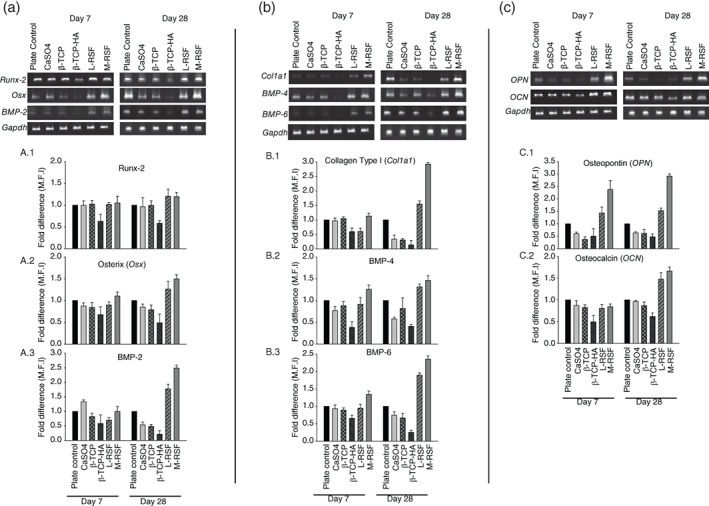
Comparison of expression of genes involved in osteoblast differentiation: Total RNA was isolated from human mesenchymal stem cells (hMSCs) cultured on plate control, calcium sulphate (CaSO_4_), beta tricalcium phosphate (β‐TCP), beta tricalcium phosphate with hydroxyapatite (β‐TCP‐HA), lyophilized‐regenerated silk fibroin (L‐RSF), and microparticle‐regenerated silk fibroin (M‐RSF). This RNA was converted to cDNA and amplified using RT‐PCR. The samples were run on agarose gel and visualized. Panel (a) shows the expression of early differentiation markers (Runx‐2, Osterix, BMP‐2). (a.1–a.3) Shows densitometric analysis of these bands. Panel (b) shows expression of middle stage markers (Collagen type I, BMP‐4, and BMP‐6). (b.1–b.3) Shows the densitometric analysis of these bands. Panel (c) shows expression levels of late differentiation markers (OPN, OCN) and (c.1) and (c.2) show densitometric analysis of these bands. Densitometric analysis was carried out using Image J software and expression levels were normalized with *GAPDH*. Data are represented as Mean ± SD (*n* = 3). **p < 0.01, ***p < 0.001, NS = not significant

**FIGURE 6:**
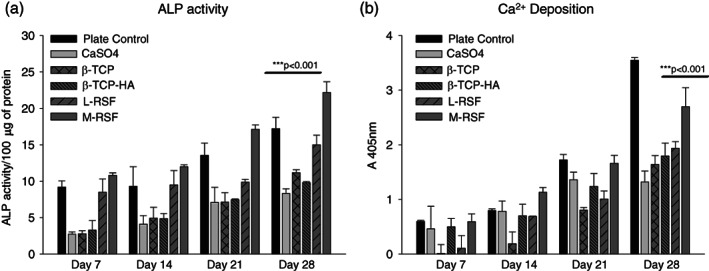
Estimation of alkaline phosphatase (ALP) and calcium deposition. Human mesenchymal stem cells (hMSCs) were seeded on calcium sulphate (CaSO_4_), beta tricalcium phosphate (β‐TCP), beta tricalcium phosphate with hydroxyapatite (β‐TCP‐HA), lyophilized‐regenerated silk fibroin (L‐RSF), and microparticle‐regenerated silk fibroin (M‐RSF). Cells were cultured in osteogenic media and incubated at 37°C, 5% CO_2_ for 28 days with media change at every 48 h. ALP activity and Ca^2+^ deposition were measured at Days 7, 14, 21, and 28. Data expressed as mean ± SD (*N* = 3) ***p < 0.001

In vivo, under physiological condition, MSCs differentiate into osteoblasts, upon exposure to appropriate microenvironment. This process is known as osteoblastogenesis, and has various stages, from commitment to terminal differentiation into osteoblasts. Various factors such as material used for construction of scaffolds, microarchitecture, bulk porosity, pore size, and matrix stiffness play a key role during the differentiation.^25^, ^26^, ^27^, ^28^ Assessment of hMSC differentiation into osteoblasts would provide crucial insights in applicability of these materials for bone void filling application. We assessed and compared the ability of SF scaffolds to support growth and differentiation of hMSCs into osteoblast, while benchmarking with commercially available ceramic bone void fillers based on CaSO_4_, β‐TCP, and β‐TCP‐HA. Proliferation was quantified by MTT assay (Figure 4), and osteoblast differentiation was quantified at three levels: (1) analysis of expression of genes and transcription factors involved in osteoblastogenesis, (2) alkaline phosphatase (ALP) activity, and (3) Ca^2+^ deposition. The genes and transcription factors were chosen to span early to late differentiation markers. ALP is a conventional and widely accepted marker indicative of osteoblast presence and Ca^2+^ deposition is the end event, leading to transformation of an osteoblast into an osteocyte, while depositing calcium.^29^ The results of gene expression studies are summarized in Figure 5 and the results of ALP activity and Ca^2+^ deposition are summarized in Figure 6.

Earlier reports suggested that cells of the same lineage behave differently in different micro‐environments provided by the scaffolds. The chemical, morphological, and mechanical characteristics of the scaffold also influence the differentiation of the stem cells.^25^ Between L‐RSF and M‐RSF, M‐RSF has better mechanical properties (wet compression modulus ~18 MPa) than L‐RSF scaffold (wet compression modulus ~3 MPa) (Table S1). These scaffolds also have vastly different microarchitectures (Figure S1). These experiments allowed us to compare the performance of scaffolds based on the same material, which are differentiated by micro‐architecture, porosity, and mechanical properties on osteoblast differentiation while benchmarking the performance with commercially available ceramic‐based bone void fillers.

#### 2.2.1 Proliferation estimation by MTT assay

During the 28 days of experimental duration, hMSCs proliferate and also differentiate into osteoblasts via osteoblastogenesis. The process of osteoblastogenesis includes intermediate cell types of osteoprogenitor cells and pre‐osteoblasts, which eventually differentiate into a mature osteoblast. All types of cells (hMSCs, osteoprogenitor cells pre‐osteoblasts, and mature osteoblasts) are present on the scaffold, under osteogenic stimulus and were estimated using MTT assay. Therefore, MTT assay gave an account of all viable cells present on the scaffold at a given time point. hMSCs cultured on L‐RSF and M‐RSF exhibited proliferation as measured by increase in absorbance at 570 nm on Days 7, 14, 21, and 28. Similar trend was observed when hMSCs were cultured on CaSO_4_, β‐TCP, and β‐TCP‐HA indicating all scaffolds supported cell proliferation. At Day 7, CaSO_4_ showed the lowest number of cells. The number of cells were found to be comparable for β‐TCP, β‐TCP‐HA, and L‐RSF scaffolds. The proliferation of cells was found to be equivalent in β‐TCP and L‐RSF for all the time points studied. It is also important to note here that compared to all other scaffolds, cells seeded on M‐RSF showed statistically significant higher proliferation (which includes hMSCs, osteogenic cells, and osteoblasts) at all‐time points of the study (Figure 4).

#### 2.2.2 Gene expression studies of osteoblast differentiation markers

Scaffolds ability to support osteoblastogenesis was evaluated by measuring levels of physiological markers of osteoblast differentiation such as Col a1, OPN, Runx‐2 (early to late markers), and osteocalcin (OCN), Osterix (OCX), bone morphogenic proteins (BMPs) (late markers).^29^, ^30^ BMP‐2, ‐4, and ‐6 play a crucial role in recruitment, proliferation, and differentiation of osteoblasts. Expression of all these genes was examined by reverse transcriptase polymerase chain reaction (RT‐PCR).^31^ Figure 5 summarizes the results of expression of markers by RT‐PCR on the agarose gel and respective densitometric analyses.

hMSCs cultured on L‐RSF and M‐RSF scaffolds showed time‐dependent increase in all the differentiation markers, whereas hMSCs cultured on CaSO_4_, β‐TCP, and β‐TCP‐HA did not show time‐dependent increase in expression levels of these markers. In fact, a decline was observed in expression levels of OPN, Runx‐2, Osterix, BMP‐2, BMP‐4, BMP‐6, and Collagen type 1 in cells cultured on β‐TCP and β‐TCP‐HA scaffolds. These scaffolds supported growth and differentiation during early timepoints (Figure 5(a)) but did not support sustained proliferation and differentiation throughout the experiment (Figure 5(b,c)). These results also corroborate with the ALP activity and Ca^2+^ deposition data (Figure 6(a,b)), where no significant time‐dependent increase in expression was observed in ceramics‐based scaffolds. Increased expression levels of osteogenic markers on both L‐RSF scaffolds and M‐RSF could be attributed to their similar chemical nature (SF). However, the increase in expression was more pronounced in M‐RSF than in L‐RSF scaffold.

#### 2.2.3 ALP expression and Ca^2+^ deposition

Osteoblasts are the main cell type involved in new‐bone formation. Osteoblasts actively participate in matrix synthesis and bone mineralization. Two key physiological markers of differentiation of hMSCs into osteoblast are ALP expression and Ca^2+^ deposition. Expression of these markers indicates presence of matured osteoblasts on the scaffolds.

##### ALP expression

ALP levels are measured as the secreted protein (Figure 6(a)). Detectable ALP activity was seen from Day 7 through Day 28, in the cells cultured on all scaffolds. From the data, it is evident that ALP expression was similar within all the ceramic‐based scaffolds indicating that they support osteoblast differentiation with equal efficiency. Both the silk scaffolds showed significantly higher ALP expression as compared to ceramic‐based scaffolds. Between M‐RSF and L‐RSF scaffolds, M‐RSF showed better ALP expression.

##### Ca^2+^ deposition

Calcium deposition is an important indicator of terminal differentiation of hMSC's into osteoblasts. Mature osteoblasts deposit calcium and eventually become osteocytes. hMSCs cultured on CaSO_4_, β‐TCP, β‐TCP‐HA and L‐RSF and M‐RSF showed time‐dependent increase in Ca^2+^deposition (Figure 6(b)), with hMSCs cultured on M‐RSF showing significantly higher Ca^2+^ deposition at all time points. Ca^2+^ deposition was similar in all other control scaffolds.

Both Ca^2+^ and ALP expression were higher in cells cultured on M‐RSF as compared to L‐RSF scaffolds. This is an interesting finding as M‐RSF and L‐RSF scaffold are chemically similar and differ only in terms of the micro‐architecture.

## 3 DISCUSSION

Bone void fillers are used to fill cavities in the bone and these fillers support and accelerate the formation of new bone. To successfully perform this function, a bone void filler must have several characteristics. In addition to biocompatibility, a bone void filler must support cell adhesion, migration, proliferation, and differentiation of hMSCs. In this work, we have compared the safety and efficacy of currently used calcium ceramic‐based bone void fillers with that of SF‐based fillers by performing a series of in vitro experiments that assess the cell–biomaterial interactions critical for bone regeneration.

Cytocompatibility of all the scaffolds was comparable as seen from the direct and extract contact cytotoxicity assays. Further, no significant differences were observed in cell adhesion on the scaffolds. Cell–biomaterial interactions mediated through various integrins expressed by the cells, supported cell attachment. The marginally lower cell adhesion for M‐RSF scaffold as compared to the L‐RSF scaffold can be attributed to the lower surface area for cell attachment due to lower bulk porosity. Additionally, all the scaffolds were found to be immunologically inert as inflammatory response was comparable for all scaffolds. This result agrees with the reported literature.^22^

Primary function of a bone void filler is to support new bone formation, which involves attachment, proliferation, and differentiation of hMSC's. This differentiation of the hMSC's into osteoblasts is influenced by the chemical, structural, and mechanical cues provided by the scaffold. Therefore, an important objective of this work was evaluation of the extent of hMSC's differentiation, when seeded on these various scaffolds.

**FIGURE 7:**
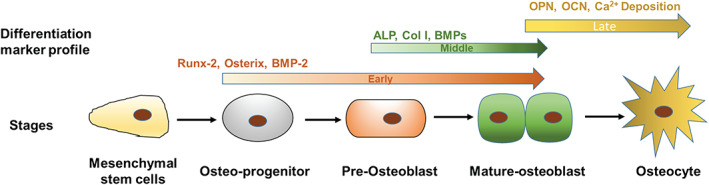
Schematic representation of stages of differentiation of mesenchymal stem cells into osteocytes: This figure depicts stages and cell types during differentiation of human mesenchymal stem cells (hMSCs) into mature osteoblasts and markers expressed at each stage of differentiation

The differentiation of a stem cell into a mature osteoblast and eventually an osteocyte is depicted in Figure 7. This involves the formation of several intermediate cells such as osteoprogenitor cells and pre‐osteoblasts.^31^, ^32^ Mature osteoblasts start depositing calcium, and embed themselves in the deposited calcium, to become osteocytes. This process is tightly regulated by expression of several markers and/or transcription factors characterizing the stage of differentiation.^33^, ^34^ This journey from a stem cell to mature osteoblast is called osteoblastogenesis. Runx‐2 is a master regulator of osteoblastogenesis and its expression leads to lineage commitment of hMSC into osteoblastogenesis. Expression of growth factors like BMP's and transcriptional regulators and factors like OCN, OPN, Osx further push the cell toward maturation as osteoblast. ALP secretion is initiated from pre‐osteoblast stage and continues in the mature osteoblasts as well. In our work, we monitored the expression of these markers in hMSC's seeded on various scaffolds at two time points, Days 7 and 28 (refer Figures 4 and 5)..^30^, ^31^, ^32^, ^33^, ^34^, ^35^

hMSCs cultured on all scaffolds supported proliferation. It must also be noted that the proliferation was found to be the lowest on the CaSO_4_ scaffold. The β‐TCP, β‐TCP‐HA, and L‐RSF scaffolds showed comparable proliferation. In spite of having marginally lower cell adhesion as compared to all other scaffolds, the M‐RSF scaffold showed enhanced cell proliferation. This indicates that the proliferation of cells on the scaffold is not only influenced by the chemical characteristics of the scaffolding material but also significantly influenced by the mechanical and structural cues provided by the scaffold.^28^, ^36^ Very low porosity has been shown to adversely affect transport of fluid and nutrients. Also, very high porosities result in higher fluid velocity and therefore affects cellular proliferation. Thus, there is an optimum porosity required for maximum cell proliferation. This observation of increase in cell proliferation with decrease in porosity has also been reported by Chen et al..^37^ The relationship of pore size with cell proliferation and differentiation has also found to be nonmonotonic.^37^, ^38^ Murphy et al. demonstrated that pore sizes of the order of 150 μm show highest cell proliferation, while further increase in pore size results in an initial decrease in cell proliferation. Thus, the higher porosity, large pore size, and pore interconnectivity in L‐RSF are not optimum for cell proliferation. Higher cell proliferation on M‐RSF scaffold as compared to L‐RSF scaffold also results in higher number of cell–cell contacts, which has found to be a major factor influencing the differentiation of hMSC's into an osteoblast. The cell–cell direct contact results in rapid transduction of cell signaling molecules.^37^

A stem cell takes cues from its micro‐environment at different levels, which ultimately determines its differentiation fate. These include cues from soluble factors like growth factors, interactions with extracellular matrix and mechanical stimuli. In our experiments, soluble factors are unchanged as we used osteogenic culture media. Also, all experiments in this study are carried out in vitro; therefore, there are no interactions with extracellular matrix (ECM).^29^ The microarchitecture, including mechanical properties was distinct for all scaffolds (described in detail in Supporting Information). A time‐dependent enhancement in the gene expression of the markers was seen in the cells cultured on all bone void fillers. As can be seen in the data summarized in Table 1, the expression of all early, early‐to‐mid, and early‐to‐late markers was found to be the lowest for β‐TCP‐HA scaffold. For most markers, the expression was found to be comparable for CaSO_4_ and β‐TCP scaffolds. The L‐RSF scaffold showed expression levels higher than these ceramic scaffolds and the M‐RSF scaffold outperformed all the scaffolds by at least 2× to 3× expression levels. Correspondingly, the ALP expression and calcium deposition or mineralization was also better supported by SF scaffolds as compared to other bone void fillers, with M‐RSF showing significantly better performance as compared to L‐RSF.

Mechano‐transduction is a process that converts the mechanical stimuli from the scaffold stiffness into a chemical response. The stiffness of a scaffold is a key “passive” mechanical cue that affects stem cell differentiation.^29^, ^39^ We have used scaffolds with similar mechanical properties (CaSO_4_ and M‐RSF), which have displayed completely varied differentiation profile. On the other hand, M‐RSF and L‐RSF scaffolds which are based on same material, but have different microarchitecture, also have diverse differentiation profile. This points to the importance of coexistence of structural and mechanical properties, in addition to chemical characteristics for optimum functioning of a bone void filler. Also, a general notion that the mechanical properties near to the native tissue, will perform well in supporting new tissue formation is validated, since M‐RSF has mechanical properties comparable to cancellous bone.

Thus, it can be concluded that from a materials perspective, SF is more suitable for bone void filling applications as compared to all the ceramic‐based scaffolds. Also, the M‐RSF scaffold performs significantly better than the L‐RSF scaffold demonstrating that pore size and pore architecture, porosity and mechanical properties of the scaffold play crucial role in cell differentiation and would therefore have an effect on new bone formation. These in vitro data must be further validated and supported by in vivo animal studies in appropriate models. Further clinical studies using the M‐RSF scaffold would validate the findings of this work.

## 4 MATERIALS AND METHODS

### 4.1 Materials

#### 4.1.1 Scaffold preparation

##### Silk scaffolds

We used two different types of silk scaffolds. Both the scaffolds are made of SF protein and have been processed in unique ways.^40^, ^41^ This leads to different mechanical characteristics and structural properties. Mechanical properties, pore size, and porosity were measured as described in the study by Nisal et al..^19^ Both scaffolds are described in detail below and their characteristics have been summarized in Table S1.

###### M‐RSF scaffolds

M‐RSF is a scaffold of fused microspheres (that are crystalline and solid) exhibiting ~40% bulk porosity, interconnected pore structure and compressive modulus equivalent of cancellous bone (described in greater detail in Refs. 19, 40, 41). In brief, microparticles of SF were prepared by using a two‐solvent system. The microparticles are monodispersed and have diameter of around 500–600 μm. These microparticles are highly crystalline (Crystallinity index = 1.6 ± 0.1) and nonporous. The scaffold was prepared by fusing these SF microparticles in a cylindrical mold using aqueous SF solution. The compression modulus of the scaffolds is 70 ± 6 MPa (dry) and 18 ± 2 MPa (wet) and have a bulk porosity of ~40%–44%.

###### L‐RSF scaffolds

These scaffolds are made by lyophilizing regenerated silk fibroin (RSF) solution. To make these scaffolds, 200 μl of 4% of aqueous SF solution was poured in a 96‐well plate and frozen at −80°C for 16 h. These frozen scaffolds were lyophilized and later sterilized by autoclaving before use. Scaffolds thus prepared have poor mechanical properties (compression modulus [dry] 10 ± 2 MPa and [wet] 3 ± 2 MPa as compared to M‐RSF scaffolds, lower crystallinity (Crystallinity index = 1 ± 1.1) and have very high bulk porosity (90%–95%).

##### Ceramics‐based bone void fillers

Various ceramic‐based commercial bone void fillers were used as is or prepared as per protocols described by the manufacturer. We selected CaSO_4,_ ^16^ β‐TCP,^17^ and a composite of β‐TCP with HA^18^ for our studies as these are commercially used materials for bone void filling applications. Digital micrographs of these scaffolds and a scanning electron microscopic image have been included in Supporting Information (Figure S1B). Details of the chemical composition and physical properties of these ceramic‐based bone void fillers are listed in Table S1.

#### 4.1.2 Cell lines

Mouse fibroblast cell‐line L929 and mouse macrophage cell line RAW 264.7 were purchased from National Center for Cell Sciences (NCCS) (Pune, Maharashtra, India) and maintained in Dulbecco's Modified Eagle's Medium (DMEM) (HiMedia, Mumbai, India) with 10% FBS (Gibco, Grand Island, NY). Bone marrow‐derived human mesenchymal stem cells (hMSCs) were purchased from Lonza (Walkersville, MD). hMSCs were maintained in Iscove's Modified Dulbecco's Medium (IMDM) (HiMedia) and 10% MSC‐FBS (Gibco, Grand Island, NY).

### 4.2 Methods: Biocompatibility testing

#### 4.2.1 Cytotoxicity testing

##### Direct contact method

L929 cells were seeded in tissue culture plate at a seeding density of 10,000 cells/well and cultured in DMEM + 10% FBS for 24 h at 37°C, and 5% CO_2_. After 24 h of incubation, media were replenished and CaSO_4_, β‐TCP, β‐TCP‐HA, L‐RSF, and M‐RSF scaffolds were carefully placed on L929 monolayer and incubated further for 24 h. Post incubation, material was gently removed and MTT assay was performed. Absorbance was measured at 570 nm and % viability was calculated using the following formula:

$$\%\text{viability}=\left(A_{T}/A_{\mathrm{PC}}\right)*100$$

$$A_{T}=\text{Absorbance of test}\;\mathrm{at}\;570\;\mathrm{nm}$$

$$A_{\mathrm{PC}}=\text{Absorbance of tissue culture plate control}\;\mathrm{at}\;570\;\mathrm{nm}.$$

Organo‐tin stabilized polyurethane (Specialty Innotech, Pune, India) scaffolds were used as positive control, while high‐density polyethylene (Sigma‐Aldrich) scaffolds were used as negative control for cytotoxicity. Data are expressed as % viability ± SD with the plate control as a reference 100%. Three independent experiments with triplicates were performed.

##### Extraction method

For preparation of extract, 32 mg of ceramic blocks/scaffolds were incubated in 200 μl DMEM + 10% FBS for 24 h at 37°C. L929 cells were seeded in culture plate at a seeding density of 10,000 cells/well and cultured in DMEM + 10% FBS for 24 h at 37°C, 5% CO_2._ After 24 h, control wells were replenished with complete media, while tests wells were treated with extracts of respective scaffolds. Cells were incubated further in the presence of extract for 24 h. Postincubation, MTT assay was performed. Absorbance was measured at 570 nm and % viability was calculated as mentioned above.

Thirty percent of DMSO was used as positive control for cytotoxicity while tissue culture plate was used as negative control. Data expressed as % viability ± SD. Three independent experiments were performed in triplicates.

#### 4.2.2 Cellular adhesion

L929 cells were seeded onto plate control, CaSO_4_, β‐TCP, β‐TCP‐HA, L‐RSF, and M‐RSF scaffolds. For plate control seeding density was 10,000 cells/well while for scaffolds, the seeding density was adjusted as per the volume of scaffold. Cells were seeded to achieve final seeding density of 2000 cells/mm^3^. Cells were cultured in DMEM + 10% FBS for 24 h at 37°C and 5% CO_2_. Postincubation these scaffolds were washed with 1X PBS and stained with 2% crystal violet for 30 min at 37°C. After incubation, cells were washed twice with 1X PBS. Crystal violet was extracted in 5% SDS and absorbance was measured at 595 nm. % cellular adhesion was calculated by following formula:

$$\%\text{Cellular adhesion}=\left(A_{T}/A_{\mathrm{PC}}\right)*100.$$

$$A_{T}=\text{Absorbance of test}\;\mathrm{at}\;595\;\mathrm{nm}.$$

$$A_{\mathrm{PC}}=\text{Absorbance of tissue culture plate control}\;\mathrm{at}\;595\;\mathrm{nm}.$$

Assay was performed in triplicate for each sample. Data are expressed as % cellular adhesion ± SD.

#### 4.2.3 Assessment of in vitro inflammatory response

In vitro inflammatory response was assessed by using an assay based on RAW 264.7 cells (procured from NCCS, Pune, India). Cells were seeded at a density of 10,000 cells/well. These cells were exposed to scaffolds for time points Days 2, 4, and 7. Media were replenished every 48 h. Expression of pro‐inflammatory cytokines such as tumor necrosis factor alpha (TNF‐α) and interleukin 1 beta (IL‐1β) was estimated by semi‐quantitative reverse transcriptase polymerase chain reaction (RT‐PCR) at Days 2, 4, and 7 post‐treatment. Positive control was included by giving 1000 ng/ml lipopolysaccharide treatment (LPS, *Escherichia coli*, Sigma‐Aldrich) and cells without any treatment (plate control) were used as negative control. *b‐actin* was the internal control of a housekeeping gene. Refer Table S2 (supporting information for mRNA, cDNA, PCR analysis and primer sequences).

### 4.3 Efficacy of scaffolds in supporting differentiation of hMSC's into osteoblasts (bone cells)

Effectiveness of the scaffolds in supporting proliferation and differentiation of hMSC's was evaluated. The following parameters were monitored (a) cell proliferation, (b) RT‐PCR of early to late differentiation markers, (d) ALP activity assay, and (c) calcium deposition. In brief, hMSCs were trypsinized and seeded onto collagen‐coated wells (plate control), and all scaffolds. Seeding density for plate control was 8000 cells/well. For scaffolds, seeding density was adjusted according to the volume of the scaffold. Cells were seeded to achieve final seeding density of 2000 cells/mm^3^.

Desired cell number was suspended in 10 μl of media which was seeded on each scaffold placed in a nonadherent 96‐well cell culture plate. These plates were maintained at 37°C, 5% CO_2_ for 30 min to allow cell adhesion. After incubation, IMDM with 10% MSC FBS was added to each well and further incubated at 37°C, 5% CO_2_ for 24 h. Scaffolds without cells were considered as blank. After 24 h, IMDM media were replaced by Stem pro‐osteogenic differentiation media (Thermo‐Fischer Scientific, Massachusetts). Media were changed every 48 h. Conditioned media were collected for estimation of ALP activity while scaffolds and plate controls were used for measurement of cell proliferation, gene expression, and calcium deposition.

#### 4.3.1 Estimation of cell proliferation

Proliferation of cells cultured on all types of scaffolds was assessed by MTT (3‐(4,5‐dimethylthiazol‐2‐yl)‐2,5‐diphenyltetrazolium bromide) assay on Days 7, 14, 21, and 28. Conditioned media were removed and scaffolds (with cells and blank) were incubated with 0.5 mg/ml MTT (Sigma‐Aldrich, St. Louis, MO, USA) for 4 h in dark at 37°C and 5% CO_2_. The blue purple formazan crystals were dissolved in DMSO and absorbance was measured at 570 nm. Each assay was performed in triplicate where *n* was at least 3. Data were expressed as mean absorbance at 570 nm ± SD.

#### 4.3.2 Gene expression of osteogenic differentiation markers by RT‐PCR

Total RNA was extracted from hMSCs seeded and cultured on plate control, CaSO_4_, β‐TCP, β‐TCP‐HA, L‐RSF and M‐RSF on Days 7 and 28 by TRIzol method (Invitrogen Life Technologies, Carlsbad, CA) as mentioned above. Concentration of RNA was measured using Nano‐drop. cDNA was prepared from 200 ng of total RNA using Verso cDNA synthesis kit according to manufacturer's instructions. The PCR conditions used and annealing temperatures for each gene are as described in Table S3. PCR products were resolved on 1.2% agarose gel and visualized using SYBR gold stain (Invitrogen) on Bio‐Rad, Molecular Imager, ChemiDox™ XRS+ imaging system. Band intensity was then analyzed using ImageJ software. Values were normalized using GAPDH (housekeeping gene control).

#### 4.3.3 Estimation of ALP activity

ALP activity was determined using colorimetric ALP activity assay kit (Abcam, Cambridge, UK). Briefly, hMSCs were cultured in well plate (plate control) and on scaffolds as described above. Conditioned media were collected on Days 7, 14, 21, and 28. ALP activity was estimated from conditioned media as per manufacturer's instructions. Assays were performed in triplicates where *n* was at least 3. Data were normalized to protein concentration and expressed as units of ALP/100 μg of protein.

#### 4.3.4 Estimation of calcium deposition by Alizarin Red S staining

For estimation of calcium deposition, cells/scaffolds with cells were washed with PBS and fixed in 10% formalin for 15 min. Cells/scaffolds were washed three times with PBS and stained with 2% Alizarin Red S solution at room temperature for 30 min with intermittent shaking. Postincubation cells/scaffolds were washed with distilled water.

Post washing, water was removed, and Alizarin Red S was extracted in 10% acetic acid at room temperature for 30 min. After incubation, cells/scaffolds were heated at 95°C for 10 min. Post incubation cells/scaffolds were incubated in ice for 15 min. This solution was then centrifuged at 12,000 rpm for 15 min at room temperature. Supernatant was transferred in 96‐well plate and absorbance was measured at 405 nm. Assays were performed in triplicates and each assay was performed at least thrice to validate the observations. Data were expressed as mean ± SD.

## 5 CONCLUSION

Synthetic bone void fillers are increasingly being used to fill cavities in the bone. In this work, we used a set of in vitro tests to assess the safety and efficacy SF‐based bone void fillers vis‐a‐vis the currently used calcium ceramic‐based bone void fillers. We selected two types of SF scaffolds that differed in their pore architecture, bulk porosity, and mechanical properties such as compression modulus. It can be concluded here that all scaffolds, irrespective of the chemical composition and physical characteristics, were noncytotoxic and supported cellular adhesion. The scaffolds did not elicit any inflammatory response as monitored through the expression of cytokines such as TNF‐α and IL‐1 β. We also monitored the expression of several differentiation markers and/or transcription factors to evaluate the ability of the scaffolds to support bone tissue regeneration. All the scaffolds were found to support early stage of differentiation of hMSC's. The expression of differentiation markers was found to be lowest in the β‐TCP‐HA, while the CaSO_4_ and β‐TCP scaffold performed marginally better. The L‐RSF scaffold showed expression levels higher than these ceramic scaffolds and the M‐RSF scaffold outperformed all the scaffolds by at least 2x to 3x expression levels. These results suggest that SF is a superior material for bone void filling applications. Further, the structural attributes of the scaffold such as bulk porosity, pore size, and compression modulus also significantly influence the performance in bone void filling applications.

## AUTHOR CONTRIBUTIONS

**Rucha Deshpande:** Conceptualization; data curation; formal analysis; investigation; software; visualization; writing‐original draft; writing‐review & editing. **Swati Shukla:** Conceptualization; data curation; formal analysis; funding acquisition; investigation; project administration; resources; supervision; validation; visualization; writing‐original draft; writing‐review & editing. **Raeesa Sayyad:** Formal analysis; resources. **Shalmali Salunke:** Formal analysis; resources. **Anuya Nisal:** Conceptualization; data curation; formal analysis; funding acquisition; investigation; project administration; resources; supervision; validation; visualization; writing‐original draft; writing‐review & editing. **Premnath Venugopalan:** Conceptualization; formal analysis; funding acquisition; visualization; writing‐review & editing.

### PEER REVIEW

The peer review history for this article is available at https://publons.com/publon/10.1002/btm2.10221.

## Supporting information

**Figure S1** (A) Photographs of CaSO_4_, β‐TCP, β‐TCP‐ HA, L‐RSF and M‐RSF scaffolds. and (B) scanning electron microscopic images of CaSO_4_, β‐TCP, β‐TCP‐ HA, L‐RSF and M‐RSF scaffolds.

**Table S1** Properties of various silk and ceramic scaffolds used in the study

**Table S2** Primer sequences and product size of inflammatory markers and housekeeping control.

**Table S3** Describes that primer sequences and product size of osteoblast differentiation markers and housekeeping control.

## Data Availability

Data available on request from the authors
