# Supplementary material for: Silk fibroin and ceramic scaffolds: Comparative in vitro studies for bone regeneration
Source: Bioeng Transl Med. 2021 Apr 8;6(3):e10221. doi: 10.1002/btm2.10221 (PMC8459602; doi:10.1002/btm2.10221)
Supplement: Supplementary file 1 — Figure S1 (A) Photographs of CaSO4, β‐TCP, β‐TCP‐ HA, L‐RSF and M‐RSF scaffolds. and (B) scanning electron microscopic images of CaSO4, β‐TCP, β‐TCP‐ HA, L‐RSF and M‐RSF scaffolds. [file BTM2-6-e10221-s002.pdf]

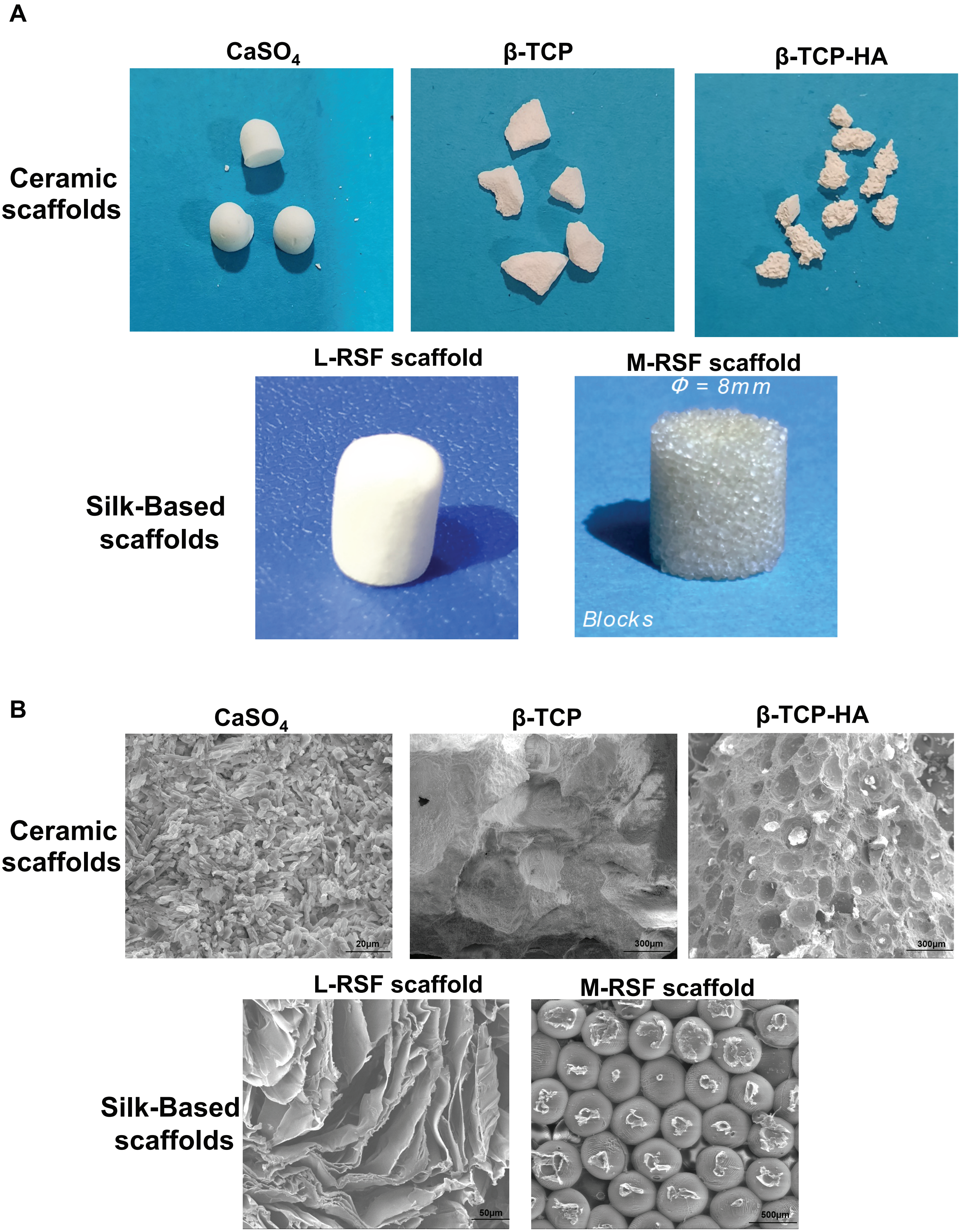
A
CaSO4
β-TCP
β-TCP-HA
Ceramic scaffolds
L-RSF scaffold
M-RSF scaffold
Φ = 8mm
Silk-Based scaffolds
Blocks
B
CaSO4
β-TCP
β-TCP-HA
Ceramic scaffolds
20μm
300μm
300μm
L-RSF scaffold
M-RSF scaffold
Silk-Based scaffolds
50μm
500μm
