# Supplementary material for: Silk fibroin and ceramic scaffolds: Comparative in vitro studies for bone regeneration
Source: Bioeng Transl Med. 2021 Apr 8;6(3):e10221. doi: 10.1002/btm2.10221 (PMC8459602; doi:10.1002/btm2.10221)
Supplement: Supplementary file 2 — Table S1 Properties of various silk and ceramic scaffolds used in the study [file BTM2-6-e10221-s003.pdf]

Table S1: Properties of various ceramic and silk scaffolds used in the study

Property	Ceramic scaffolds			Silk scaffolds	
	**$CaSO_4$**	**β-TCP**	**β -TCP-HA**	**L-RSF**	**M-RSF**
**Compression modulus**	~80 MPa (Dry) ~70 MPa (Wet)	~ 5MPa	Not available	~ 10MPa (Dry) ~ 3MPa (Wet)	~70 MPa (Dry) ~ 18 MPa (Wet)
**Porosity**	10-12%	60-70%	60-70%	90-95%	40-44%
**Pore size**	Randomly packed crystals < 5 µm	100-500 µm (macropores) ≤10 microns (micropores)	300-600 µm (macropores) ≤10 microns (micropores)	10-200 µm (Random pores)	0-275µm
**Pore structure**	Random pores	Random pores	Random pores	Random pores	Interconnected pores between micro-particles
