# Supplementary material for: Silk fibroin and ceramic scaffolds: Comparative in vitro studies for bone regeneration
Source: Bioeng Transl Med. 2021 Apr 8;6(3):e10221. doi: 10.1002/btm2.10221 (PMC8459602; doi:10.1002/btm2.10221)
Supplement: Supplementary file 3 — Table S2 Primer sequences and product size of inflammatory markers and housekeeping control. [file BTM2-6-e10221-s004.pdf]

Table S2: Primer sequences and product size of inflammatory markers and housekeeping control.

Sr. No	Gene name	Primer sequence	Annealing temperature	Product size
1.	*Tnfa*	F5’GCGAGGTGGAACTGGCAGAAG3’	68°C	362 bp
		R5’ GGTACAACCCATCGGCTGGCA3’		
2.	*Il1b*	F5’TCATGGATGATGATGATAACCTGCT3’	60°C	502 bp
		R5’CCCATACTTTAGGAAGACACGGATT3’		
3.	*b-actin*	F 5’TGGAATCCTGTGGCATCCA3’	62°C	315 bp
		R 5’TAACAGTCCGCCTAGAAGCA3’		

The PCR conditions used for 30 cycles of amplification were as follows:

PCR Model used: Eppendorf Master cycler, realplex^2, ep gradient S

Stage	Temperature	Time	Number of cycles
Initial denaturation	95°C	3 min	1
Denaturation	95°C	30sec	30
Annealing	Temperature as mentioned in TableS1	45 sec	
Extension	72°C	1 min	
Final Extension	72°C	5 min	1
Hold	4°C	-	-
