# Supplementary material for: Silk fibroin and ceramic scaffolds: Comparative in vitro studies for bone regeneration
Source: Bioeng Transl Med. 2021 Apr 8;6(3):e10221. doi: 10.1002/btm2.10221 (PMC8459602; doi:10.1002/btm2.10221)
Supplement: Supplementary file 4 — Table S3 Describes that primer sequences and product size of osteoblast differentiation markers and housekeeping control. [file BTM2-6-e10221-s001.pdf]

Table S3: Describes that primer sequences and product size of osteoblast differentiation markers and housekeeping control.

Sr. No.	Gene name	Primer sequence	Annealing Temperature	Product size
1.	*OCN*	F5’ GAGCCCCAGTCCCCTACCCG3’	60°C	114bp
		R5’ AGCGCCGATAGGCCTCCTGA3’		
2.	*OPN*	F5’ CCATGTGGACAGCCAGGACTCCA3’	62°C	142bp
		R5’ TGATGGCAGGTCCGTGGGAA3’		
3.	*Osx*	F5’ CCCCTACCCAGCTCCCCACC3’	60°C	167BP
		R5’ CACTCCCCCCATAAGGTGG3’		
4.	*Runx-2*	F5’ ACCTGCCACCACCCTACCCC3’	62°C	123bp
		R5’ CCCCCGGCACCATGGGAAAC3’		
5.	*Col1a1*	F5’ GTGGAAACCCGAGCCCTGCC	62°C	129bp
		R5’ CAGACGGGACAGCACTCGCC3’		
6.	*BMP-2*	F5’ ACACAAACAGCGGAAACGCCT3’	60°C	107bp
		R5’ GATACCCCGGGGGAGCCACA3’		
7.	*BMP-4*	F5’ AGGAGGCTGGATATAGTTTCTG3’	60°C	326bp
		R5’ TTCTCAGGGATGCTGCTGAGGTTA 3’		
8.	*BMP-6*	F5’ CCTGGGATGGCAGGACTGGA 3’	62°C	229bp
		R5’ AGGTCTGCACAATCGCGTGG 3’		
9.	*Gapdh*	F5’ GAAGGTGAAGGTCGGAGT 3’	60°C	226bp
		R5’ GAAGATGGTGATGGGATTTC 3’		

mRNA, cDNA and PCR analysis.

Total RNA was extracted from RAW 264.7 cells on plate control, cell exposed to scaffolds and to LPS treatment, by TRIzol method (Invitrogen Life Technologies, Carlsbad, CA, USA). In brief, cells were incubated with 500µl TRIzol reagent overnight at 4°C. 200µl of chloroform

was added to the cell lysate and the suspension was centrifuged at 10,000rpm for 16min at 4°C. Aqueous layer was collected, 350µl of iso-propanol was added, and solution was centrifuged at 10,000rpm for 15min at 4°C. Pellet was washed with 70% ethanol, air dried and dissolved in nuclease free water. Concentration of RNA was measured using Nano-drop (ND-1000, UV/Vis spectrophotometer, Nano-drop technologies, USA). cDNA was prepared from 200ng of total RNA using Verso cDNA synthesis kit (Thermo-Fisher Scientific, Waltham, MA, USA) according to manufacturer's instructions. The PCR conditions used and annealing temperatures for each gene are as described in Table S1. PCR products were resolved on 1.2 % agarose gel and visualized using SYBR gold stain (Invitrogen) on Biorad; Molecular Imager, ChemiDox™ XRS+ imaging system. Band intensity was then analyzed using ImageJ software. Values were normalized using *b-actin* (housekeeping gene control). Primers were designed using Gene Runner software.

The PCR conditions used for 30 cycles of amplification were as follows:

PCR Model used: Eppendorf Master cycler, realplex^2, ep gradient S

Stage	Temperature	Time	Number of cycles
Initial denaturation	95°C	3 min	1
Denaturation	95°C	30sec	30
Annealing	Temperature as mentioned in TableS2	45 sec	
Extension	72°C	1 min	
Final Extension	72°C	5 min	1
Hold	4°C	-	-
